# A new animal model for delayed osseous union secondary to osteitis

**DOI:** 10.1186/s12891-015-0816-7

**Published:** 2015-11-19

**Authors:** Lars Helbig, Thorsten Guehring, Svenja Rosenberger, Adriana Ivanova, Kathrin Kaeppler, Christian Alexander Fischer, Arash Moghaddam, Gerhard Schmidmaier

**Affiliations:** Clinic for Orthopedics and Trauma Surgery, Center for Orthopedics, Trauma Surgery and Spinal Cord Injury, Heidelberg University Hospital, Schlierbacher Landstrasse 200a, 69118 Heidelberg, Germany; Klinik für Unfallchirurgie und Orthopädie, BG Unfallklinik Ludwigshafen, Ludwig-Guttmann-Strasse 13, 67071 Ludwigshafen, Germany

**Keywords:** Animal model, Rat, Delayed osseous union, Osteitis, Biomechanical testing, Micro-CT

## Abstract

**Background:**

The treatment of infection-related delayed bone unions is still very challenging for the orthopedic surgeon. The prevalence of such infection-related types of osteitis is high in complex fractures, particularly in open fractures with extensive soft-tissue damage. The aim of this study was to develop a new animal model for delayed union due to osteitis.

**Methods:**

After randomization to infected or non-infected groups 20 Sprague–Dawley rats underwent a transverse fracture of the midshaft tibia. After intramedullary inoculation with staphylococcus aureus (10^3^ CFU) fracture stabilization was done by intramedullary titanium K-wires. After 5 weeks all rats were euthanized and underwent biomechanical testing to evaluate bone consolidation or delayed union, respectively. Micro-CT scans were additionally used to quantitatively evaluate the callus formation by the score of Lane and Sandhu. Blood samples were taken to analyze infectious disease markers (day 1, 14 and 35).

**Results:**

Biomechanical testing showed a significant higher maximum torque in the non-infected group 5 weeks postoperatively compared with the infected group (*p* < 0.001). According to the Lane and Sandhu score a significantly higher callus formation was found in the non-infected group (*p* < 0.001). Similarly, the leucocyte count in the infected group was significantly higher than in the non-infected group (*p* < 0.05).

**Conclusions:**

Here we have established a new animal model for delayed osseous union secondary to osteitis. The animal model appears to be appropriate for future experimental studies to test new therapeutic strategies in these difficult to treat bone healing complications.

## Background

The treatment of non-healing bone defects, especially those secondary to infection, is a great challenge for the orthopaedic surgeon. Treatment often lasts months to years and involves radical and repeated surgical debridement in combination with intensive antibiotic treatment [1]. Recurrent infections are not uncommon and can lead to the loss of the affected extremity, which has enormous professional, social, financial, and familiar consequences for patients [1].

The incidence of infections and osteomyelitis after complex fractures is high, especially in open fractures with large tissue damage [2, 3]. Staphylococcus aureus is responsible for approximately 55 % of osteomyelitis cases [4].

In the last few years, surgeons have begun to use BMPs in combination with autologous human mesenchymal stem cells (MSC) to treat non-unions [5, 6]. The development of an effective treatment concept could result in a more confident treatment strategy and ultimately an overall improvement in patients’ quality of life. In addition, it would decrease the time and costs of treatment (e.g., a reduction in hospital stay, follow-up treatment, and resources). The goal of this study was to develop and establish a new animal model of delayed fracture healing in order to investigate innovative treatment concepts secondarily.

## Methods

Implant-related infection in rats was induced according to a previously established protocol [7]. S. aureus was used for inoculation (subspecies aureus Rosenbach) obtained from LGC Standards, Wesel, Germany (ATCC 49230). This strain is sensitive to flucloxacillin, gentamicin, and erythromicin, but resistant to penicillin.

### Preparation of bacterial inoculum

For each surgery a new inoculum of S. aureus (ATCC 49230) was prepared by an overnight culture in BD Brain Heart Infusion (BHI) (Becton Dickinson Diagnostic Systems, Heidelberg, Germany) solution. Cells were pelleted and washed twice in phosphate-buffered saline (PBS). CFU (colony-forming unit) counts were determined by plating serial dilutions on blood agar. The final bacterial suspension in PBS consisted of 10^3^ CFU/10 μl [8].

#### Implant

Titanium Kirschner wires, 0.8 mm in diameter (Synthes GmbH, Umkirch, Germany), were used for osteosynthesis.

The following treatment groups were examined:

Group I (infected; *n* = 10): K-wire osteosynthesis and infected with 10^3^ CFU Stapylococcus aureus;

Group II (non-infected; *n* = 10): K-wire osteosynthesis with no infection;

For biomechanical testing a comparison with the contralateral tibiae of group I and group II was done (see Fig. 2).

#### Animals, operative procedure, and fracture model

All experiments were approved by the Animal Experimentation Ethics Committee of Karlsruhe (35–9185.81/G-171/11). Twenty female, 6-months-old Sprague–Dawley rats (Harlan- Winkelmann, Borchen, Germany) were operated on for study purpose. Surgery was performed under general anesthesia by weight-adopted intraperitoneal injection of Xylazin 2 % (Medistar®; 12 mg/kg body weight) and Ketamaninhydrochlorid (Ketavet; 100 mg/ml; 80 mg/kg body weight) after sedation of the animals with isoflurane in a sedation-box.

Animals were prepared for surgery as follows:

The right hind leg was shaved, depilated, and disinfected with alcohol. The animals were placed on sterile drapes, the bodies were covered with sterile sheets. Skin and fascia at the proximal tibial metaphysis were incised over 5 mm in length. With a k-wire, a 1 mm hole was drilled through cortical and cancellous bone to access the medullary cavity at the proximal metaphysis. The medullary cavity was bluntly reamed with a steel Kirschner wire (1.0 mm diameter) followed by reaming with a 0.8 mm Kirschner wire in order to reach the narrowing distal part of the tibial cavity. After removal, 10 μl of bacterial suspension containing 10^3^ CFU/10 μl of S. aureus (ATCC 49230) were injected with a 50-μl micro syringe (Hamilton®, IL, USA) for contamination of the medullary cavity according to study groups [7].

After contamination, the right tibia and fibula were fractured with an established fracture device [9]. The right leg was externally rotated and placed on two rounded bolts at a 20 mm distance. The leg was fixed on a stop-plate at a variable distance. A third bolt was placed 2–4 mm above the tibiofibular junction. A weight (650 g) was fixed on this bolt with a removable pin. The drop-down distance of the weight was 15 cm. An impulse of *p* = 1.12 Ns generated a closed transverse fracture of the tibia in the midshaft (AO 42-A3, respectively) and the fibula. After closed reduction, the tibiae underwent an intramedullary stabilization with a 0.8 mm titanium Kirschner wire (Synthes GmbH, Umkirch, Germany). The excess parts of the Kirschner wires were cut off at the site of entry. Soft tissue was irrigated, and skin and fascia were sutured in a single knot technique (Resolon® 3/0 Ethicon, Norderstedt, Germany).

### Follow-up

All animals received buprenorphine (0.03 mg/kg bw; Temgesic®) as analgesic medication perioperatively. Animals were followed up for 5 weeks and were then sacrificed. All procedures were carried out under inhalation anesthesia with isoflurane (Forene®). On the day of surgery and regularly throughout the observation period, body weight and body temperature were measured. The clinical condition of the animals was evaluated.

#### μCT scan Evaluation

μCT scans were taken throughout the observation periods on day 0 and week 5, respectively. μCT scans were performed with a SkyScan 1076 in vivo x-ray microtomograph (SkyScan n.v., Aartselaar, Belgium) as previously described [10]. The tibiae were scanned using an isotropic voxel size of 19 μm using energy settings of 50 kV and 200 μA, a 0.5-mm aluminum filter, and eight repeated scans. Image reconstruction was performed (SkyScan NRecon package v. 1.5.1.4) by correcting for ring artifacts and beam hardening (20 %). Following image reconstruction, the individual fracture lines were identified by simultaneously viewing multiple orthogonal slices (SkyScan DataViewer v. 1.4). The region of interest for each bone was determined approximately 3 mm proximal and distal to the fracture line (150 images). Bridging of the fracture callus was evaluated by two independent observers according to the modified x-ray score of Lane and Sandhu [11]. The ratings for each sub-item were given according to the degree of bone formation and union. In details a completed bone formation was rated with 4 points, and no bone formation with 0 points. The degree of bone union was rated according to the clearance of the fracture line (4 points = complete clearance with no detectable fracture line, 2 points = partial clearance, and 0 points = no clearance clearly visible fracture line).

#### Blood and serum analyses

Blood and serum samples (0.5 mL) were taken from the cauda vein on days 0, 14, and week 5. The blood samples were analysed for routine laboratory parameters (blood count, leucocytes count, and C-reactive protein) as previously described [9].

#### Body weight and body temperature

Rectal body temperature was measured and body weight was determined with a precision scale on days 0, 7, 14, 21 and 28. Further indications for local or systemic infections were evaluated.

### Sacrifice

Under general anesthesia, 3 ml of blood were drawn from the cauda vein for final blood and serum analyses. The animals were sacrificed with CO_2_ in a sedation box. The right tibiae of the hind legs were dissected under sterile conditions. The entire soft tissue was removed from bones.

#### Microbiological evaluation

The Kirschner wires were explanted, rolled over nutrient agar (BD™ Columbia Agar, Becton Dickinson, Heidelberg, Germany), and placed in 2.5 ml sterile TSB. Agar plates and TSB were incubated at 37.8 °C. After 24 h, CFU on agar plates were counted and bacterial growth in the tryptic soy solution was evaluated (cloudy: positive growth; clear: no growth) [12].

#### Mechanical testing

**Fig. 1:**
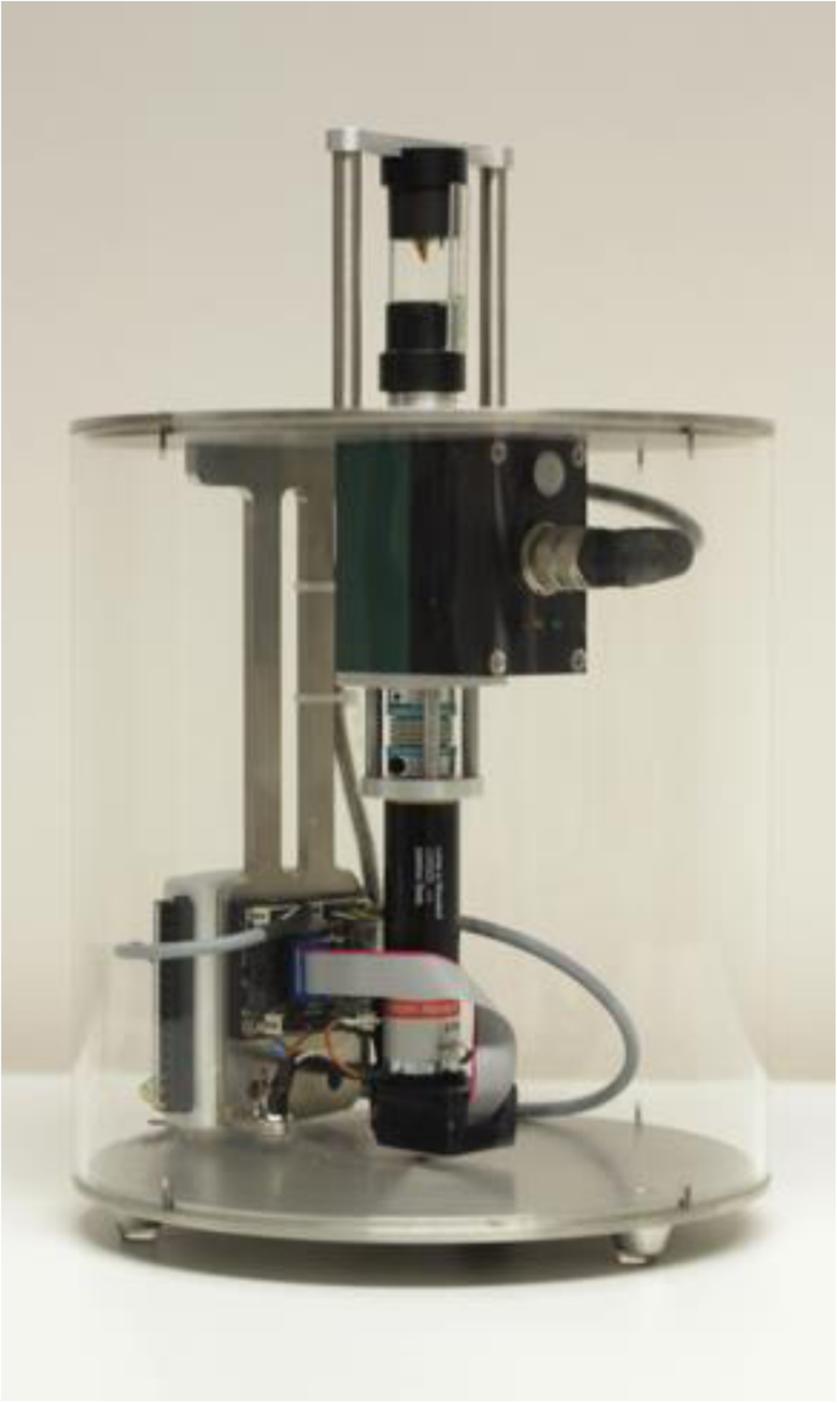
Picture of the biomechanical testing machine

At 5 weeks after fracture, all animals were killed and both tibiae were dissected free from soft tissue for biomechanical torsional testing. After dissection of the bones, the proximal and distal ends were embedded into two embedding molds with bone cement (Beracryl, Troller, Fullenbach, Germany). Each embedding mold was connected to a pivoted axis. A linear, constant feed rate, initiated by a materials testing machine (Fig. 1) was loaded by a lever attached to one of the pivoted axes. The bone was preloaded with an axial force of 5 N and a constant linear propulsion (v = 2 mm/min) was applied by the testing machine. The translation of the materials-testing machine was transformed to a uniform torsional movement. The free axis was connected with a strain gauge (*F*max = 50 N; HBM, Germany) that determined the level of torsion and transferred the data to a calculator.

#### Statistics

Primary outcome was measured according to the maximal torque in Nm. Secondary outcome measures were the modified x-ray score according to Lane and Sandhu and the leucocyte count. Complete data sets were available for 20 animals. Mean and standard deviation (SD) were calculated for continuous, median, and interquartile ranges for ordinal variables. Association between continuous and discrete variables was tested with Student’s *t* test. Data of the outcome variables and confounders were tested in a one-way analysis. Comparison of the data was performed using one-way analysis of variance (ANOVA) for independent samples and balanced with the Bonferroni-Holm Test if more than two groups were compared. In the case of abnormal distribution of LAR-values in this study, median, interquartile range, and Mann–Whitney-*U* test results were calculated. All tests were two-sided and a *p* value ≤ 0.05 was considered significant. Statistical analysis was performed using GraphPad Prism version 4.00 for Windows, GraphPad Software, San Diego California USA.

## Results

### Failure parameters

Two animals died due to anesthesia immediately after operation for reasons that could not be detected. The dropout animals were replaced.

### Mechanical testing

**Fig. 2:**
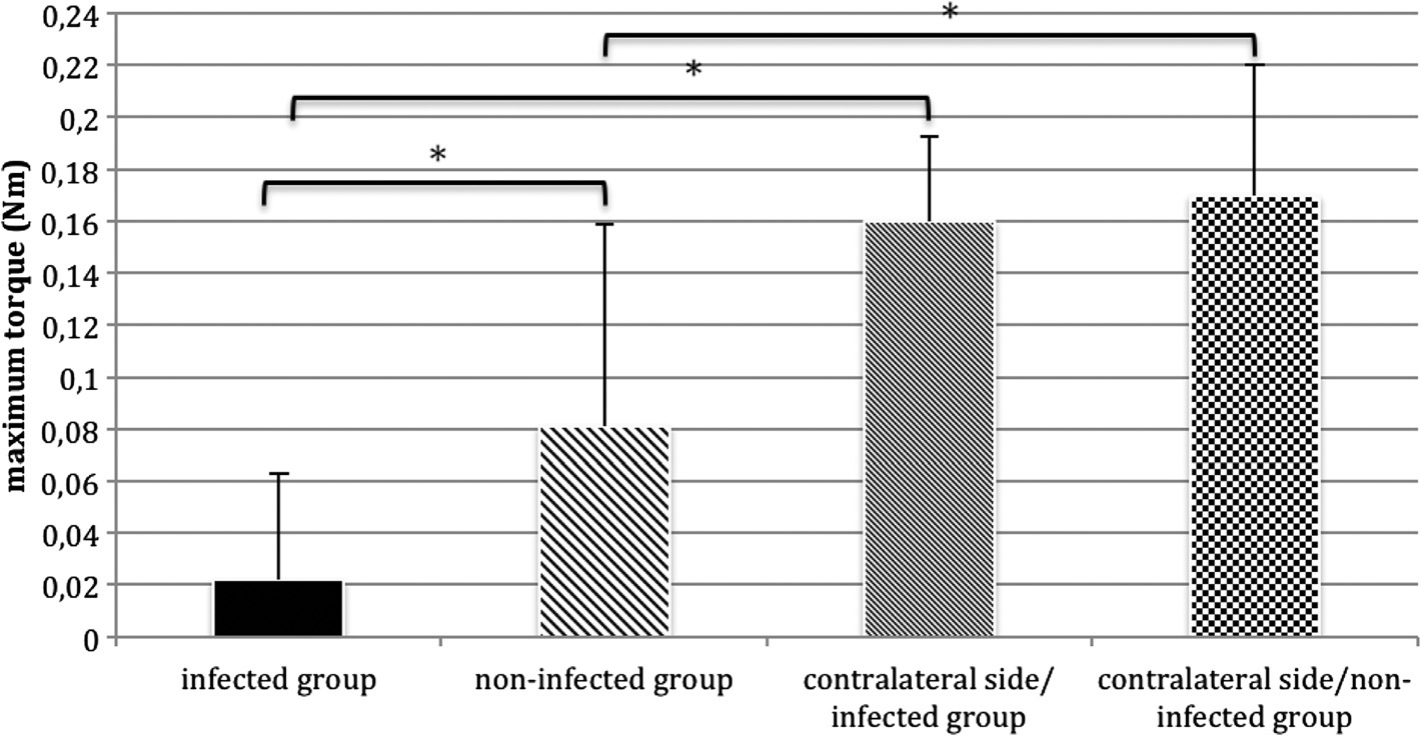
Maximum torque (Nm) of the fractured non-infected group II (*n* = 10) was significantly higher compared to the fractured infected group I (*n* = 10) (* *p* < 0.001, ANOVA). Similarly, statistically significant differences were found between the fractured side of both groups (infected and non-infected groups) and the contralateral side (* *p* < 0.001, ANOVA). *Asterisk* indicates significant difference in comparison to the other groups; * *p* < 0.001; mean+/− standard deviation

After sacrifice the intramedullary implants were carefully removed for biomechanical investigation. The K-wires could be removed easily in all cases, with no differences detected between the infected and non-infected groups. The tibiae of group I (infected group) and group II (non-infected group) were compared with the non-fractured contralateral tibiae (contralateral side/infected group; contralateral side /non-infected group; Fig. 2). Maximum torque (Nm) of the fractured side in the non-infected group (Group II; *n* = 10) was significantly higher than the fractured side in the infected group (Group I; *n* = 10) after 5 weeks (*p* < 0.001, ANOVA). In details the tibiae of the fractured non-infected group II showed an average maximum torque of 0.081 +/− 0.078 Nm compared to 0.022 +/− 0.041 Nm in the fractured infected group I (Fig. 2). Maximum torque (Nm) of the fractured side was significantly lower than the contralateral side in group I and II (*p* < 0.001, ANOVA). The differences between the non-infected and infected group on the non-fractured contralateral side showed no statistical significance (*p* = 0.379; ANOVA).

### μCT scan examinations and modified x-ray score according to Lane and Sandhu

**Fig. 3:**
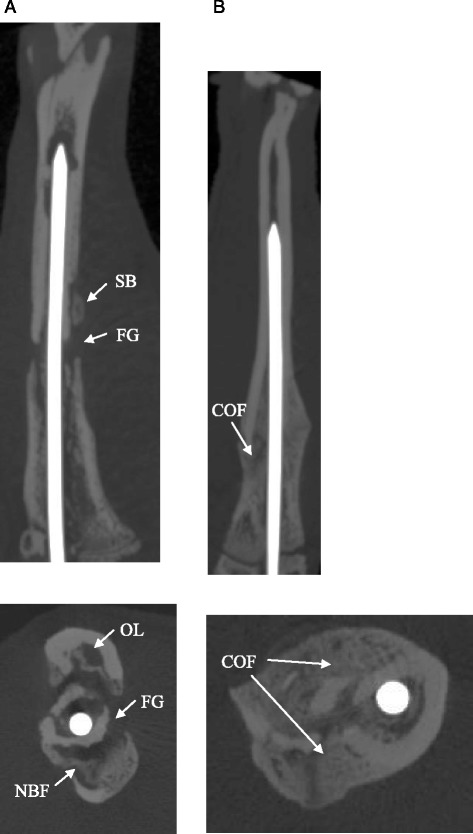
**a** X-rays (lateral and axial) of the right tibia of a Sprague–Dawley rat (infected group) 35 days after fracture and intramedullary stabilization with titanium Kirschner wires. The fracture gap (FG) is still detectable. X-rays of the right tibia clearly depict signs of infection with osteolysis (OL), periosteal new bone formation (NBF) and sequestered bone (SB). **b** X-rays (lateral and axial) of the right tibia of a Sprague–Dawley rat (non-infected group) 35 days after fracture and intramedullary stabilization with titanium Kirschner wires. The progression of consolidation of fracture (COF) in the non-infected group is clearly recognizable compared to the infected group. No radiographic signs of infection can be observed in animals of the non-infected group

**Fig. 4:**
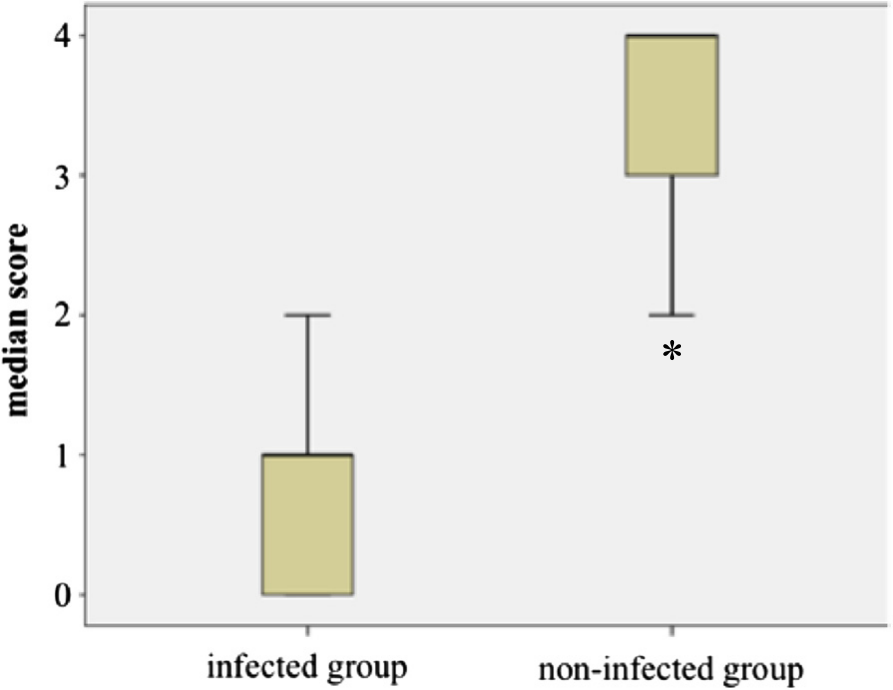
Outcome of the modified x-ray score of Lane and Sandhu 35 days post op. X-ray score of Lane and Sandhu in the non-infected group (*n* = 10) (median = 4) is significantly higher than in the infected group (*n* = 10) (median = 1); * *p* < 0.001

Compared with the non-infected group (II; *n* = 10), the infected group (I; *n* = 10) showed clearly reduced consolidation of the fractures after 5 weeks (Fig. 3a and b). The fracture gap was not bridged after 5 weeks in ten of ten animals. The tibiae of the non-infected group were bridged completely in nine of ten animals after 5 weeks (group II). The results of the modified x-ray score according to Lane and Sandhu showed highly significant differences between the two groups. The median score was four (range 2–4) in the non-infected group (II) and 1 (range 0–2) in the infected group (I) (*p* < 0.001; Mann–Whitney-*U* test) (Fig. 4).

### Blood and serum analyses

**Fig. 5:**
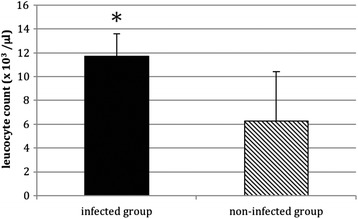
Results of blood analyses 2 weeks post op. A significantly higher leucocyte count is seen in the infected group (*n* = 10) (11.71 +/− 1.89 × 10^3^ /μl) compared to the non-infected group (*n* = 10) (6.25 +/− 4.17 × 10^3^ /μl); * *p* < 0.05; mean+/− standard deviation

No significant differences (*p* > 0.05, ANOVA) for C-reactive protein were detectable between the infected and non-infected group and throughout the entire study period (data not shown). But the leucocyte count 14 days postoperatively showed significant differences between the two groups. The leucocyte count in the infected group (*n* = 10) (11.71 +/− 1.89 × 10^3^ /μl) was significantly higher than in the non-infected group (*n* = 10) (6.25 +/− 4.17 × 10^3^ /μl) (*p* < 0.05, ANOVA) (Fig. 5). At the remaining time-points no significant differences for leucocyte count were found.

### Microbiological evaluation / cultures of implants

After sacrifice, Staphylococcus aureus could be cultured from all implants of group I (infected group; *n* = 10) following incubation in TSB. The corresponding agar plates revealed massive bacterial growth far above 1000 CFU.

All implants of group II (non-infected group; *n* = 10) remained sterile in culture.

### Body weight and body temperature

No significant differences of body weight and body temperature were detected between groups. Body weight moderately decreased in the infected and non-infected group during the first 2 weeks, but the animals gained weight continuously in the next 3 weeks. Body temperature remained stable in all groups during the 5 weeks (data not shown).

## Discussion

Posttraumatic osteitis along with associated impaired fracture healing is still a serious complication in orthopedics and trauma surgery. Treatment is difficult and long lasting. Patients are confronted with substantial and often irreversible health problems, which have socioeconomic consequences [1, 13]. Especially patients with open fractures [2, 3] or with extensive soft tissue damage have a higher risk of infection and therefore greater chance of impaired healing [14, 15]. Depending on the patient risk profile, this percentage lies between 10 and 30 % [1].

Delayed—not due to infection—fracture healing (hypertrophic non-union) can be successfully treated by established operative methods for treating non-unions, e.g., dynamized intramedullary nailing, switching to a thicker intramedullary nail, autologous cancellous bone graft, or additional stabilization of the fracture with an anti-rotation plate [14, 16]. The method of choice for biological activation of fracture healing with threatening or manifested atrophic non-union is the application of autologous cancellous bone grafts [16]. If this method fails, the treatment of the non-union becomes problematic [5].

A bacterial infection of the fracture can occur due to the primary injury, iatrogenic causes during operative care, or hematogenic factors in the course of therapy [2, 15, 17–20]. Depending on findings, the removal of the osteosynthesis material or an extensive debridement with possible far reaching bone resection may be necessary [2]. The optimal treatment for non-union and delayed union secondary to osteitis involves both surgery and medical treatment [21, 22]. A radical surgical debridement in combination with stable osteosynthesis should be followed by an extended period of appropriate antibiotic treatment.

S. aureus is one of the most common bacterial pathogens in orthopedic and traumatological infections after open fractures. Furthermore, animal model experiments in the literature have been conducted mainly with S. aureus [7, 23]. For this reason, we have chosen this pathogen in our newly developed animal model of delayed osseous union secondary to osteitis.

In this study, we have developed a new animal model for delayed osseous union secondary to osteitis through the use of both μCT scan examinations and biomechanical investigations. To our knowledge this is the first animal model of delayed osseous union secondary to osteitis that closely monitors the course of delayed osseous union according to biomechanical investigations and μCT scan examinations. Together with the previously shown biomechanical testing [8] and the μCT scan examinations, this model will be appropriate for future studies assessing the efficacy of new osteosynthesis techniques with bone substitutes and growth factors aimed at accelerating bone healing. In addition, it appears to be valuable for future experimental studies testing new therapeutic strategies for delayed osseous union [24].

Many alternative models with both their advantages and disadvantages have been described in the literature. Models of impaired fracture healing can be divided into models of delayed union or nonunion (atrophic and hypertrophic), segmental defects, and fracture-related osteitis [25].

Many different bone defect animal models of osteitis have been described in the literature [26–29]. Windolf et al. [27, 28] used a mice model with locking plate fracture stabilization, and here bone healing was evaluated by radiological, immunological and histological evaluation. Schindeler et al. [29] established a rat open fracture model by inoculation with Staphylococcus aureus. In this study the antibiotic CSA-90 was tested alone and in combination with recombinant human bone morphogenetic protein 2 (rhBMP 2), respectively. An open femoral osteotomy with periosteal stripping was performed to generate an open fracture. Radiographic analysis using a μCT and histological examination were used to evaluate the amount of new bone formation. Chen et al. [26] used a rat femur model with a segmental bone defect by creating a chronic infection with stabilization by a plate and k-wires. The bone healing was assessed by μCT, histological evaluation and torsional failure testing. In all these animal models fracture was created using an osteotomy [26–29]. In the current study we have introduced a model in which the tibiaewere fractured with an established fracture device [30], which might allow an appropriate simulation of a “typical” closed fracture. In opposite to an osteotomy model the soft tissue remained closed in our model. However own pre-experiments showed that with our fracture device the individual fracture pattern was more complex and difficult to predict. Consequently we carefully aimed to create the fracture as standardized as possible to obtain a transverse fracture type (42-A3/AO). On the other hand one disadvantage of our model could be the relatively poor reproducibility as compared to an osteotomy model, which might explain a poorer healing tendency of fracture models [24]. Furthermore, only one other study has evaluated the bone healing using a torsional failure testing [26], despite the fact that a biomechanical stability testing is probably one of the most important parameters to evaluate bone healing in complicated fracture healing [24].

Despite the growing knowledge on the mechanisms of fracture healing, delayed healing and non-union formation remain major clinical challenges. Animal models are needed to study the complex process of normal and impaired fracture healing and to develop new therapeutic strategies [24].

## Conclusions

A new animal model for delayed osseous union secondary to osteitis has been developed. Such standardized animal models are essential for future experimental studies to evaluate new therapeutic strategies in these difficult to treat bone healing complications.

## Abbreviations

BHI: brain heart infusion
BMP: bone morphogenetic protein
CFU: colony-forming unit
CT: computer tomography
COF: consolidation of fracture
FG: fracture gap
MSC: mesenchymal stem cells
NBF: new bone formation
OL: osteolysis
PBS: phosphate-buffered saline
S. aureus: staphylococcus aureus
SB: sequestered bone
TSB: tryptic soy broth
